# MAGENTA: a Multinational patient survey assessing the Awareness, perceptions and unmet needs in GENetic Testing and counselling among patients with breAst cancer

**DOI:** 10.3389/fonc.2024.1380349

**Published:** 2024-05-14

**Authors:** Sarah Powell, Marta Artigas, Irina Borovova, Poorva Gadiya, Alice Hsu, Ranjit Kaur, Lisa Kidd, Denise Rosenfeld, Mai Mohamed Saeed, Evelin Scarelli, Magdy Waheeb Youssef

**Affiliations:** ^1^ Pink Hope, Narrabeen, NSW, Australia; ^2^ Independent Researcher, Buenos Aires, Argentina; ^3^ Russian Association of Oncology Patients “ZDRAVSTVUY!”, Moscow, Russia; ^4^ Nag Foundation, Pune, India; ^5^ Independent Researcher, Taipei, Taiwan; ^6^ Breast Cancer Welfare Association Malaysia, Selangor, Malaysia; ^7^ Reach to Recovery International, Towson, MD, United States; ^8^ Advanced Breast Cancer Global Alliance, Lisbon, Portugal; ^9^ Victorian Department of Education, Beaconsfield Primary School, Beaconsfield, VIC, Australia; ^10^ Independent Researcher, Mexico City, Mexico; ^11^ Independent Researcher, Cairo, Egypt; ^12^ OncoGuia Institute, São Paulo, Brazil; ^13^ Medical Affairs, AstraZeneca International, Cairo, Egypt

**Keywords:** breast cancer, patient survey, genetic testing, genetic counselling, BRCA

## Abstract

**Objective:**

Genetic testing and counselling are critical in assessing breast cancer risk and tailoring treatment strategies. However, several barriers hinder patients from opting for genetic testing/counselling, leading to fewer than one-third of patients undergoing testing and even fewer being offered counselling. A granular understanding of these barriers is essential in overcoming them.

**Methods:**

A multinational survey developed by patient authors was conducted in 9 countries, to identify the specific local/regional barriers. The survey question pathway was individualized, based on responses to prior questions. Percentage responses to a response option were calculated based on the total number of respondents to that question. Chi-square tests were used to assess the significance of the results, if applicable.

**Results:**

The final analysis set (FAS) included 1,176 respondents, with a subset of this responding to all questions. In the FAS, 63% of respondents had undergone testing. Among those who got tested, 70% were offered testing. Among untested respondents, only 40% were offered the test but eventually did not get tested. In the tested population, 44% received counselling, which was significantly higher than 7% (p<0.00001) in the untested group. Among those reporting on awareness, 71% reported awareness level between ‘very low’ and ‘moderate’ prior to cancer diagnosis. Most respondents (71%) agreed that all breast cancer patients should undergo testing before treatment initiation. However, Asian patients were less likely to endorse this view compared to respondents from other regions (25% vs ≥50%; p<0.00001). A higher proportion of tested respondents were ‘very willing’ to get their family members tested (44%) versus untested respondents (11%), with relatively higher willingness among Australian (77%) and Russian respondents (56%), the regional variation being statistically significant (p<0.00001).

**Conclusions:**

Critical gaps remain in the access, awareness and perceived value of genetic testing and counselling, with regional variance or difference between the tested and untested groups. Most patients are not offered counselling, which may be associated with the low uptake of testing. Strategic action is needed to drive policy-shaping and improve access to testing and counselling, including raising patient awareness and improving patient experience for better treatment outcomes.

## 1 Introduction

Breast cancer is one of the leading cancers in women, with more than 2 million new cases diagnosed globally in 2020 (1). Having a close blood relative with a history of breast cancer and the presence of inherited mutations in genes like the BReast CAncer genes 1 (*BRCA1*) and 2 (*BRCA2*) raises breast cancer risk in approximately 5%–10% of women (2, 3). Mutations in genes other than *BRCA1/2* (for example *ATM*, *PTEN*, *TP53*, *CHEK2*, *CDH1*, *PALB2*, *STK11*) also present with a considerably high likelihood of developing breast cancer, but with a lower frequency than *BRCA1/2* (2, 3).

Approximately half of the individuals carrying such genetic mutations do not present with family history (2). Therefore, breast cancer screening relying primarily on family history may preclude early diagnosis of some high-risk women, potentially compromising treatment outcomes (2). Genetic testing complements proactive self-surveillance of breast cancer and may significantly improve patient outcomes through early identification of patients at high risk, risk-reducing surgery or individualized systemic anti-cancer treatment (4).

Cancer risk assessment through genetic testing and counselling in individuals at a high risk of hereditary or familial cancer is recommended by the National Comprehensive Cancer Network (NCCN) guidelines (v3.2023) (5). The NCCN guidelines recommend genetic testing for *BRCA1*, *BRCA2*, *CDH1*, *PALB2*, *PTEN*, *STK11* and *TP53* in women aged ≤50 years with a personal history of breast cancer. Testing is also recommended in women of any age, with personal or family history of breast cancer, undergoing specific treatments or with specific pathological or histological features of breast cancer (for example, triple-negative breast cancer) (5). The American Society of Clinical Oncology recommends testing for *BRCA1/2* mutations to all patients newly diagnosed with breast cancer, who are of ≤65 years old. *BRCA1/2* mutation testing is recommended for select patients who are >65 years, based on personal history, family history, ancestry or poly(ADP-ribose) polymerase (PARP) inhibitor therapy eligibility (6).

Pre-test and post-test counselling is recommended by the NCCN guidelines (5). Pre-test counselling should include but not be limited to knowledge-sharing on breast cancer genetic testing, goals for cancer risk assessment in family members, patient medical history, differential diagnosis, patient education on genetic inheritance patterns and preparation for the outcomes of the test. In addition, pre-test counselling should involve obtaining written informed consent, planning for the disclosure of the test results and the impact of the result on treatment strategy. Data privacy protection and legal protection from discrimination in employment or insurance coverage based on the results of the genetic test should also be discussed, along with cost and insurance coverage for testing and counselling (5). Post-test counselling should include discussion of the test results and its associated medical risks, interpretation of results in view of personal and family history of cancer, impact on treatment strategy, psychosocial support for those experiencing anxiety or distress through the process and resources for notifying family members of the test results (5).

Despite global guidelines recommending genetic testing and counselling, socioeconomic and cultural factors, lack of awareness, inadequate insurance coverage or poor knowledge of reimbursement policies, as well as insufficient healthcare resources including qualified genetic counsellors, limits the uptake of genetic testing and counselling. This is particularly true for low- or middle-income countries, where lack of infrastructure and resources compounds sociocultural and educational barriers in accessing these services. In addition to these barriers, limited local and regional statistics on the uptake of these services is a major knowledge gap which contributes to limited access of these tests.

In Asia, cost of testing, inadequate reimbursement policies, lack of awareness on the benefits of testing and social prejudices against those carrying hereditary disorders are key barriers (7–9). Similarly, in Middle-Eastern countries, poor awareness of cancer inheritance, risk and availability of testing infrastructure are the predominant challenges (10). Similar to Asia, cost and lack of insurance coverage for genetic tests are critical barriers in Latin America, along with insufficient expertise in oncogenetics among healthcare providers, lack of recognition of genetic counselling as a clinical discipline and lack of supportive healthcare policies (11). Despite national guidelines of several Latin American countries recommending genetic counselling and testing in those with a personal or family history of cancer, the availability of breast cancer genetic testing is poor and not included in public insurance schemes (11). Risk reduction interventions are recommended by regional guidelines in the case of hereditary breast cancer syndromes, but may not be covered by public health insurance or if covered, have limited accessibility due to other barriers (11).

While cost is not a significant barrier to testing in Australia due to mainstreaming of genetic testing for several breast cancer genes, the restrictive eligibility criteria result in a considerable proportion of at-risk women not receiving the benefits of genetic testing and counselling (12, 13). Absence of a clear family history due to sociocultural factors also contributes to limited uptake of genetic testing and counselling in Australia. Under-referral by doctors, limited awareness of the testing process and lack of confidence in supporting the patients through the testing process further compound the problem (12, 14).

Despite the Global Breast Cancer Initiative Implementation Framework that was announced by the World Health Organization (WHO) to close the care gap and promote equitable cancer care globally, critical gaps remains in policies, infrastructure and awareness of breast cancer genetic testing and counselling (15). Most efforts in improving breast cancer care are directed towards addressing the barriers associated with the role of healthcare professionals with limited focus on barriers pertaining to the patient’s experience. Hence, a comprehensive understanding of barriers and challenges in the breast cancer genetic testing and counselling landscape derived from patients in different regions is critical in advising strategies tailored to the region.

The Multinational survey study Assessing GENetic Testing and counselling among patients with breAst cancer (MAGENTA) was conducted for a comprehensive understanding of the breast cancer genetic testing and counselling experience and to identify the challenges encountered by patients in accessing these services. The main themes explored by this survey were arrived upon through comprehensive discussions by the ‘Genetic testing and breast cancer – Patient author steering committee’. A survey was designed to enquire on different aspects of the breast cancer genetic testing and counselling process, including awareness levels, perceived value, testing experience, the impact of testing, access to testing and reimbursement policies.

## 2 Methods

### 2.1 Survey design

The ‘Genetic testing and breast cancer – Patient author steering committee’ comprised of patient authors from 9 countries (Argentina, Australia, Brazil, Egypt, India, Malaysia, Mexico, Russia and Taiwan) who have had breast cancer. The steering committee convened on 28 April 2022 to discuss the breast cancer genetic testing and counselling landscape across geographies and patient subgroups and identify the key themes to be explored by the MAGENTA survey. In consultation with representatives from patient advocacy groups (PAGs; **Supplementary Table 1**) from their respective countries and AstraZeneca Medical Affairs and Patient Affairs representatives, the committee drafted a 38-question survey. This multiple-choice questionnaire was developed in English and translated to Arabic, Hindi, Malay, Portuguese, Russian, Spanish and Traditional Chinese, for dissemination in the countries of the respective patient authors. The survey was adapted according to local definitions of education and income levels by local PAGs and AstraZeneca medical & patient affairs representatives and disseminated with the support of the local PAGs on their social media or patient group platforms. The anonymized survey (**Supplementary Table 2**) was offered to participants through different media channels.

The question flow in the survey is shown in **Supplementary Figure 1**. Questions enquiring on the baseline demographics of the respondents (age at breast cancer diagnosis and at the time of the survey, country of origin, income level, educational attainment) and if they had undergone breast cancer genetic testing and counselling, were offered to all survey participants. Select questions enquiring on the barriers to testing, awareness levels, reimbursement policies etc., were also offered to all participants, although a response was not mandatory. For the remaining questions, the survey pathway was individualized and dependent on the response to previous questions (**Supplementary Figure 1**). As a result, apart from the baseline demographics, most questions have a unique number of total respondents. The total number of respondents to each question in this survey is denoted as ‘n_q_’ where ‘n’ is the total number of respondents to ‘q’, the question number; ‘n_q,y_’ denotes the number of respondents to question number ‘q’, who responded with the response option ‘y’ (**Supplementary Table 2** and **Supplementary Figure 1**).

### 2.2 Data analysis

All responses were included in the survey analysis excluding responses which were incomplete or if the response data were of poor quality. A response was designated as being of poor quality if the response was recorded under a duplicate identification number or IP address, if there was no response to ≥1 question on baseline demographics or to ≥1 L1 question (questions offered to all participants), or if there was a mismatch in baseline characteristics data. Percentage responses to a response option were calculated as 100 multiplied by n_q,y_/n_q_. Subgroup analysis of the survey results was performed according to region, income level, educational attainment and if the respondents had received a genetic test or not. Chi-square tests were used to assess statistical significance of the differences observed in subgroups analyzed, if applicable.

## 3 Results

### 3.1 Baseline characteristics

A total of 1,524 respondents undertook the survey with 1,176 (77.2%) of them forming the full analysis set (n_FAS_; **Supplementary Figure 2**). Respondents were equitably distributed across the various geographies surveyed (**Table 1**), with the highest recruitment from Asia [India, Malaysia, Taiwan; n_Asia_ = 472 (40.1% of n_FAS_)], followed by Latin America [Argentina, Brazil, Mexico; n_LATAM_ = 310 (26.4% of n_FAS_)], Russia [n_Russia_ = 149 (12.7% of n_FAS_)], Middle East [Egypt; n_ME_ = 141 (12.0% of n_FAS_)] and Australia (n_Aus_ = 104 (8.8% of n_FAS_)]. In the n_FAS_, median age at the time of the survey was 47 years [interquartile range (IQR): 39, 55] and at the time of diagnosis was 42 years (IQR: 35, 49). In the n_FAS_, 54.9% belonged to the low- or medium-income level, with 39.4% of the n_FAS_ preferring not to disclose their income level (**Table 1**). Most n_FAS_ respondents were open to disclosing their educational attainment level, with 87.5% having medium or advanced level of education (**Table 1**).

**Table 1:** Baseline characteristics of survey respondents.

Characteristic	N=1176
Country of origin, n (%)	
Asia	
India	45 (3.8)
Malaysia	168 (14.3)
Taiwan	259 (22.0)
Latin America	
Argentina	26 (2.2)
Brazil	205 (17.4)
Mexico	79 (6.7)
Middle East	
Egypt	141 (12.0)
**Others**	
Australia	104 (8.8)
Russia	149 (12.7)
Age group at the time of the survey, n (%)	
Median age [years (IQR)]	47 (39, 55)
18–44 years	528 (44.9)
45–64 years	556 (47.3)
65–74 years	87 (7.4)
Above 75 years	5 (0.4)
Age group at the time of the diagnosis, n (%)	
Median age [years (IQR)]	42 (35, 49)
18–44 years	657 (59.9)
45–64 years	445 (37.8)
65–74 years	72 (6.1)
Above 75 years	2 (0.2)
Income level, n (%)	
Low	293 (24.9)
Medium	353 (30.0)
High	67 (5.7)
Prefer not to say	463 (39.4)
Educational attainment, n (%)	
Low	141 (12.0)
Medium	223 (19.0)
High	628 (53.4)
Advanced	179 (15.2)
Prefer not to say	5 (0.4)
Received genetic testing	
Yes	737 (62.7)
No	439 (37.3)
Received genetic counselling	
Yes	358 (34.6)
No	543 (52.6)
I am not sure	132 (12.8)

The baseline characteristics of the respondents in the final analysis set have been compiled here.

### 3.2 Prevalence of genetic testing in surveyed respondents

**Figure 1:**
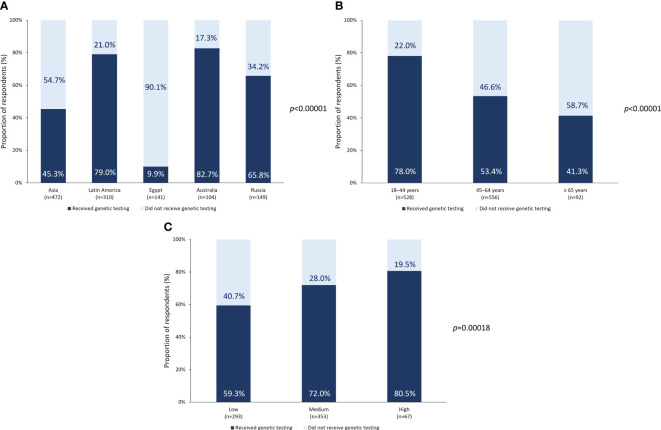
Genetic testing prevalence. Prevalence of genetic testing according to **(A)** region or country of origin, **(B)** age at the time of survey, and **(C)** income level.

Overall, 737 respondents in the n_FAS_ (62.7%) had undergone genetic testing (n_6,yes_; **Table 1**) and 439 had not (n_6,no_). Subgroup analysis according to region showed that among respondents from Australia, Latin America, and Russia, 82.7% (of n_Aus_), 79.0% (of n_LATAM_) and 65.8% (of n_Russia_), respectively underwent genetic testing (**Figure 1A**). However, only 45.3% of n_Asia_ and 9.9% of n_ME_ (Egypt) had received a genetic test. The differences in the prevalence of genetic testing according to region were statistically significant (p <0.00001; **Figure 1A**). Respondents in the age group of 18–44 years and those with a medium to high household income were also more likely to undergo genetic testing than older individuals or those with a low household income (**Figures 1B, C**).

### 3.3 Prevalence of genetic counselling in surveyed respondents

In the n_FAS_, 1,033 respondents (87.8%) responded to the question on whether they received genetic counselling (n_21_). Among these 1,033 respondents, 543 (52.6%) did not receive genetic counselling. Subgroup analysis comparing the prevalence of genetic counselling between tested and untested respondents was performed. Among the 737 respondents who underwent genetic testing. 327 (44.4%) received genetic counselling. However, among the 439 respondents who did not undergo testing, 31 (7.1%) had received genetic counselling. These results indicate two significant care gaps: (1) not all breast cancer patients who get tested receive counselling, thereby receiving an incomplete experience of the genetic testing and counselling workflow, which results in a compromised patient experience; and (2) a larger proportion of tested respondents also receive counselling, which may indicate that receiving genetic counselling may have been positively correlated with deciding to undergo a genetic test. The correlation between receiving genetic counselling and undergoing a genetic test was found to be statistically significant (p<0.00001).

### 3.4 Awareness of genetic testing and counselling and barriers to testing

Self-awareness of breast cancer genetic testing and counselling before disease diagnosis was enquired upon, along with the survey participants’ perception of awareness levels in their doctor and their community (**Table 2**). Out of the 1,061 respondents to the question [n_27(self)_], 758 (71.4%) reported a ‘moderate to very low’ self-awareness, prior to their breast cancer diagnosis, which is the combined result for response options ‘very low’, ‘low’ and ‘moderate’. Similarly, perceived community-level awareness among the 856 respondents [n_27(community)_] was reported to be ‘moderate to very low’ by 780 (91.1%) respondents. The level of awareness of genetic testing and counselling among doctors was perceived to be ‘high or very high’ by 439 (51.2%) out of 858 [n_27(doctor)_] respondents, with responses for options ‘high’ and ‘very high’ being combined.

**Table 2:** Awareness levels regarding genetic testing and genetic counselling among survey respondents, respondents’ doctor and the respondents’ community.

	Very high or high n (%)	Moderate to very low n (%)
**Self, before diagnosis (n=1061)**	303 (28.6)	758 (71.4)
**Doctor (n=858)**	439 (51.2)	419 (48.8)
**Community (n=856)**	76 (8.9)	780 (91.1)

The percentage responses to the response options in Q27 have been compiled here. The number of respondents to response options ‘very high’ and ‘high’ have been combined and the number of respondents responding with ‘moderate’, ‘low’ and ‘very low’ have been combined.

Regarding the role of their oncologist in the genetic testing process, only 134 (21.7%) out of a total of 618 respondents to the question (n_8_) reported having been referred for genetic testing, receiving an explanation about the test and the results throughout the process and using the result to inform treatment selection, by their oncologist. This implies that ~80% of n_8_ respondents received insufficient or no explanation regarding genetic testing and its implications from their oncologist. When enquired about the resources guiding their genetic testing experience beyond their oncologist or doctor (n_15_ = 616), 295 (47.9%) respondents reported relying on their genetic counsellor, 232 (37.7%) on patient support groups, 217 (35.2%) on websites, 195 (31.7%) on brochures/pamphlets and 161 (26.1%) on social media groups.

To understand the prevalence of genetic testing being offered to breast cancer patients, survey participants were asked if they had been offered genetic testing. Among the 984 respondents (n_18_), 691 (70.2%) reported being offered genetic testing by their doctor, indicating that 29.8% of respondents were not proactively offered the test. Interestingly, out of the total n_6,no_, i.e., the untested respondents, 174 (39.6%) reported having been offered testing by their doctor, but not proceeding with the test, which indicates that these respondents might have had some reservations regarding the test, despite being made aware of it.

Based on the results of the survey, the patient authors concluded that poor physician and patient awareness, the inability of physicians in providing an impactful explanation of the implications of the test and the limited proportion of physicians offering the test to their patients were probable reasons for suboptimal genetic testing and counselling rates.

### 3.5 The genetic testing and counselling experience

Among the respondents reporting on their emotions during the testing process (n_9_ = 616), 607 (98.5%) reported no regret in getting a genetic test, with the most prevalent emotions during genetic testing being anxiety (n=254; 41.2%), concern (n=128; 20.8), fear (n=123; 20.0%) as well as gratefulness (n=130; 21.1%) (**Supplementary Table 3**). Interestingly, genetic counselling was reported to be ‘very helpful’ by 211 out of 352 respondents (60.1%), implying that the emotional distress experienced during testing could potentially be tackled by support from genetic counselling.

Responding to the question regarding how they came to decide on getting a genetic test (n_13_ = 617), 338 respondents (54.8%) reported that talking to their doctor or genetic counsellor was the determining factor. Cancer diagnosis in another family member drove 58 respondents (9.4%) to decide to get a genetic test. Discussion on genetic testing was predominantly initiated by the oncologist as reported by 145 out of 294 respondents (n_25_; 49.3%), followed by the surgeon (22.8%).

Among 351 respondents (n_24_) reporting on who provided them genetic counselling, majority (206; 58.7%) reported receiving counselling from the genetic counsellor, with only 52 (14.8%) and 25 (7.1%) respondents receiving counselling from the oncologist and surgeon, respectively. Given the limited number of genetic counsellors available within the healthcare system of most low- or middle-income countries, oncologists or surgeons may need to play a more active role in providing genetic counselling. Therefore, oncologists or surgeons in countries lacking in genetic counsellors need to be empowered with sufficient training and tools to be able to offer appropriate counselling to their patients.

### 3.6 Perceived impact of genetic testing

Among 405 respondents responding to the question regarding at what point in their disease or treatment journey they were offered testing (n_19_), 197 (48.6%) reported having undergone genetic testing at diagnosis, followed by 146 respondents (36.0%), who underwent testing during treatment. This implies that genetic testing is conducted only after treatment initiation in a fairly large number of cases, and proactive surveillance is not common. Undergoing a genetic test post-diagnosis or at the start of treatment may limit the positive impact of genetic testing in informing treatment decisions. Changes in treatment strategy in response to the results of the genetic testing after starting treatment could potentially cause emotional stress to patients and their families.

The most common impact of genetic testing on treatment strategy as reported by the survey was changing from unilateral mastectomy to bilateral mastectomy (276 out of n_29_; 37.5%), followed by other treatment changes (221; 30.0%), addition of treatment pre- or post-surgery (172; 23.3%), changing from chemotherapy/radiotherapy to targeted therapy (153; 20.8%), addition of surgery pre- or post-treatment (74; 10.0%) and requirement of a second surgery (55; 7.5%). When enquired on the effect of changes in treatment strategy due to genetic testing, 319 out of n_30_ (43.3%) reported no effect. However, 264 respondents (35.8%) reported psychological stress, which could be a consequence of lack of genetic counselling and decision on getting tested too late in the treatment journey.

### 3.7 Perceived value of genetic testing

A key goal of this survey was to assess if survey respondents considered that the genetic testing and counselling processes were valuable in identifying and mitigating cancer risk in themselves and their family members. When enquired about this, out of a total of 849 respondents (n_31_), 603 (71.1%) opined that all patients diagnosed with breast cancer should undergo genetic testing before starting treatment. However, 206 respondents (24.2%) believed that only patients with a family history of breast cancer or other risk factors should undergo genetic testing. Out of 603 respondents who believed that all women with breast cancer should get tested, a majority (460; 76.3%) had undergone testing. This might indicate that the untested population bear some misgivings regarding the benefits of the test and are unlikely to recommend testing for all eligible women, irrespective of breast cancer history.

When enquired about the specific value of testing, 675 out of n_FAS_ (57.4%) believed that testing allowed surveillance and early detection of breast cancer in family members and 526 respondents (44.7%) believed that testing informed treatment decisions. The patient authors also align with these observations, based on their personal experience as well as the prevalent beliefs in their regional community.

However, subgroup analysis revealed regional variability in the perceived value of genetic testing for all women. Russia reported the highest proportion of respondents who believed that all eligible women should undergo genetic testing (102 out of 108 respondents; 94.4%), while Asia reported the lowest proportion of respondents (60 out of 237 respondents; 25.3%), for the same response (**Table 3**). The overwhelming response from Russian respondents in recognizing the value of genetic testing in all women may be due to the higher accessibility of genetic testing and its inclusion in the national reimbursement plan, which considerably reduces the out-of-pocket expenses of a patient undertaking the test.

**Table 3:** Opinion on the need for genetic testing in family members according to country or region of origin.

In your opinion, should all patients diagnosed with breast cancer undergo genetic testing first before starting treatment?	Asia n (%)	Australia n (%)	Middle East/Egypt n (%)	Latin America n (%)	Russia n (%)
	n=237	n=92	n=75	n=198	n=108
Yes	60 (25)	64 (70)	55 (73)	99 (50)	102 (94)
No, only patients with a family history or other risk factors	85 (36)	23 (25)	11 (15)	62 (31)	6 (6)
No	92 (39)	5 (5)	9 (12)	37 (19)	0
Total	237 (100)	92 (100)	75 (100)	198 (100)	108 (100)

This table shows the region subgroup analysis on whether all patients diagnosed with breast cancer should undergo genetic testing first before starting treatment. These responses were recorded for Q31.

Among tested respondents (n_6,yes_), 322 (43.7%) were ‘very willing’ to have their family members and children get tested versus 49 out of n_6no_ (11.2%), i.e. the untested respondents. However, Asia, Middle East, and Latin America reported a relatively lower proportion of respondents who were ‘very willing’ to have their family members or children tested (**Table 4**). These observations could be reflective of sociocultural factors where pre-empting a disease diagnosis may be considered unnecessary. The fear of social stigma, or the impact on legal rights or on family and personal life in the event of the diagnosis of a hereditary disease could be additional factors influencing respondents in being unwilling to have family members undergo testing. Additionally, there may be some guilt associated with being held responsible for passing on hereditary disorders. Therefore, ignorance may be considered as bliss. These hypotheses also reflect a lack of awareness of the benefits of proactive testing in cancer risk reduction and maximizing treatment benefit and call for further education and awareness.

**Table 4:** Willingness to get children or family members undertake genetic testing according to country or region of origin.

How willing would you be to have your children and other family members undergo genetic testing?	Asia (153)	Australia (81)	Middle East (23)	Latin America (310)	Russia (82)
	n=153	n=81	n=23	n=310	n=82
Very willing	33 (22)	62 (77)	1 (4)	70 (23)	46 (56)
Willing	23 (15)	11(14)	6 (26)	52 (17)	23 (28)
Somewhat willing	22 (14)	6 (7)	1 (4)	45 (15)	10 (12)
Less willing	50 (33)	2 (2)	9 (39)	62 (20)	1 (1)
Not willing	25 (16)	0	6 (26)	81 (26)	2 (2)
Total	153 (100)	81 (100)	23 (100)	310 (100)	82 (100)

This table shows the region subgroup analysis on the willingness of respondents to get their family members tested. These responses were recorded for Q14.

### 3.8 Economic barriers to accessing genetic testing and counselling

When enquired upon the main barriers to genetic testing for the respondents and their families, cost was identified as a key perceived barrier by 210 respondents (n_37_ = 425; 49.4%), followed by lack of understanding (n = 163; 38.4%) and social stigma and discrimination (n = 21; 4.9%). This trend was consistent in the regional subgroup analysis, with cost and lack of understanding being the top barriers in all regions surveyed.

However, cost was not the most prominent reason for not getting tested themselves, with 61 out of a total of 394 respondents (15.4%) identifying it as a reason for not getting tested. In fact, the most common reason for not getting tested was not being offered a test (282 respondents; 71.6%).

Among 849 respondents, only 313 (36.8%) believed they had knowledge and understanding of the reimbursement criteria for genetic testing in their country. In addition, 313 out of 850 respondents (36.8%) responded that they do not know if the cost of genetic testing is reimbursed in their country, even if they were to qualify for reimbursement.

The above observations highlight the inadequacy of appropriate national policies as well as lack of awareness campaigns educating the public and patient families about the existence of these resources, where applicable.

### 3.9 Proposed solutions to overcoming barriers to genetic testing and counselling

Majority opinion on the potential solutions to overcome the barriers in low uptake of genetic testing and counselling leaned towards updating of clinical guidelines, as advised by 121 respondents (n_38_ = 379; 31.9%), followed by public awareness programs for patients and the community (n = 108; 28.5%). The patient authors were in agreement that awareness and lack of guidelines were major challenges, that which addressed could significantly tackle the issue of low uptake of breast cancer genetic testing and counselling.

Other commonly proposed solutions included the education of healthcare professionals and facilitation of the qualifying criteria for genetic testing and counselling.

## 4 Discussion

### 4.1 A novel survey from the breast cancer patient’s perspective

Breast cancer diagnosis and treatment is a physically, emotionally and financially overwhelming experience and the benefits of genetic testing and counselling are manifold in supporting patients and families through this exhausting process. This patient author-driven survey, which directly recorded responses from more than 1000 participants across geographies and ethnic groups, demonstrated the real-world impact of genetic testing with 98.5% of respondents expressing no regret in undertaking the test. However, the testing process can be daunting for patients and their families, with a considerable proportion of respondents reporting feeling anxious, concerned or afraid. These sentiments underscore the need for genetic counselling as a highly supportive tool for patients and their families in understanding the benefits and implications of the test, as well as in managing their emotional distress including that of their support system, through the testing process.

### 4.2 Gaps remain in the prevalence of genetic testing and counselling

This survey revealed that most of the respondents (62.7%) underwent genetic testing although significant regional disparities exist in testing rates. Despite a large proportion of respondents reporting having undergone genetic testing, it is of great concern that almost 40% of breast cancer patients and their families in the regions surveyed did not undergo testing. This reflects a major gap in accurate cancer risk assessment in the community and an unmet need in providing optimal patient care. Additionally, this could reflect a gap in the dissemination of information regarding genetic testing by PAGs, leading to a considerable proportion of patients and their family members not recognizing the benefits of testing and getting tested.

In addition to suboptimal genetic testing uptake, the poor uptake rate (34.6%) for genetic counselling is a grave concern. This critical gap in genetic counselling might be contributing to some patients not seeking testing despite being offered or being made aware of it. In our view, if these patients were offered genetic counselling, they might have been able to better appreciate the benefits of the test and chosen to undertake it. This underscores the critical need in expanding genetic counselling services in the community, by way of training more personnel as genetic counsellors but also equipping physicians and surgeons with the tools to provide counselling.

### 4.3 Poor awareness among doctors is a major barrier to achieving an optimal patient experience

Non-ideal uptake rates of genetic testing and counselling may also be a consequence of poor awareness among patients, their community, as well as healthcare practitioners. Breast cancer patients and their families rely heavily on physicians for information, resources and support. However, the survey revealed that only half of the participants who responded to the question enquiring on the awareness level of their doctor believed their physicians have significantly high awareness of genetic testing. Further, fewer than a quarter of the respondents when enquired regarding the role of their oncologist in the genetic testing process felt that they had received a complete explanation of the workflow, the results and implications from their oncologist. This underscores the limited focus that oncologists have in guiding their patients through their disease timeline, due to paucity of time, knowledge or both.

The implications for this lack of awareness are acute. For example, in Latin America, where only ~40% of respondents consider their doctor to be very knowledgeable about genetic testing, receiving insufficient information following a positive genetic test makes patients feel insecure and diffident about their condition. A sense of attachment with their physician through information exchange and emotional support, as well as exchanging learnings from the experience of other patients, could provide a more personalized treatment journey and greater acceptance of their mutational status. Similarly in Russia, where patients’ perception of the doctor’s knowledge is poor, very low awareness of doctors in certain regions forces patients to seek consult in federal medical institutions and search for information themselves. Even after being recommended to undergo testing by federal medical institutions, patients struggle to find support among local physicians about where to get tested and how to interpret the results, often resorting to PAGs and social media for information, causing them considerable emotional distress.

Inadequate awareness levels of physicians or oncologists, or their inability to provide a comprehensive guide to their patients, represent an unmet need in the genetic testing experience of breast cancer patients. Physicians need to be provided with tools which would facilitate more detailed communication with patients and raise awareness as well as the comfort level of the patient and their families in taking the test. For example, in Australia, mainstreaming has been implemented that allows a clinician to order a genetic test and refer the patient to a family cancer clinic if they have a positive result, where there is a greater likelihood for the patient and their family to be provided with adequate counselling support.

One of the most cited solutions to improving the uptake of breast cancer genetic testing and counselling by survey respondents, was improving patient and community awareness. PAGs must also proactively drive community awareness, creating patient-friendly portals to share inspirational stories, educational content, and self-assessment tools. Low awareness levels among patients and community at the pre-diagnosis stage could be countered by leveraging patient support groups, genetic counsellors and informative websites on genetic testing. The utility of social media groups in spreading awareness is becoming increasingly pertinent, including in Latin American countries where awareness levels in the community or among doctors continue to be poor. Even in more developed healthcare systems such as in Australia, PAGs do not receive public funding, which limits their ability to disseminate evidence-based information to patients and their families, as well as the public. However, spreading awareness and information through social media poses a risk of spreading misinformation too. Therefore, PAGs are instrumental in disseminating accurate information, engaging healthcare practitioners for educational sessions as well as involving patients to effectively educate and support other patients, their families, and the community. Raising awareness among patients may help those who were not proactively offered genetic testing by their doctor to be informed about genetic testing and counselling, as well as address the potential concerns and fears of patients who were offered testing but did not proceed with it. Social media groups are also instrumental in creating a community for the exchange of experiences between patients and their family members, with that of other patients.

### 4.4 Value of genetic testing remains underappreciated

This survey revealed that majority of the participants recognized the benefits of undergoing testing and would recommend it to all eligible women. However, approximately a quarter of the respondents continue to hold a longstanding belief that testing is needed only in women with a family history of breast cancer. Since not all pathogenic variants in cancer-associated genes may manifest as hereditary breast cancer, these women who do not get tested bear the risk of developing cancer in the future. This observation highlights the need to educate patients and their families on the benefits of genetic testing in the early detection of cancer risk, even in the absence of a family history of breast cancer.

It is important to understand the concerns of these patients regarding proactive testing and alleviate them through community-awareness programs and active participation of healthcare practitioners and PAGs. While all patient authors agree that testing should be extended to all eligible breast cancer patients, irrespective of a family history of breast cancer, many authors recognize that local conventions and practices, particularly in Latin America, may restrict testing only to those with a family history. It is also recognized that physicians may have a narrower interpretation of the international testing guidelines, and as such may restrict offering a genetic test only to the most high-risk population, considering limited reimbursement support.

Prior experience with testing appears to have a positive impact on the perceived value of the test among the survey respondents, with tested respondents demonstrating a greater likelihood of recommending testing for their family members (44%) compared with respondents who did not undergo testing (11%). Therefore, more eligible women need to be encouraged to get tested, to induce a domino effect of testing and counselling in the community.

### 4.5 Genetic testing often begins too late in a patient’s treatment journey

This survey revealed that most patients accessed genetic testing either at disease diagnosis or during treatment. Therefore, most patients may not be able to benefit from proactive surveillance, thereby experiencing suboptimal treatment outcomes. Delaying testing might have implications in changing treatment decisions, which may cause significant psychological or financial distress. Therefore, it is important for healthcare practitioners to identify patients and families at high risk of breast cancer, as defined by the NCCN guidelines, and recommend them to undergo genetic testing prior to treatment initiation. The survey also revealed that ~30% respondents believe that updating regional clinical guidelines to incorporate genetic testing and counselling could be a potential solution in circumventing the barriers in achieving high uptake rates of these services.

### 4.6 Poor genetic counselling services represent a critical care gap

Most of the respondents who received genetic counselling found it to be a helpful service. However, this survey found that fewer than 40% of respondents received genetic counselling, highlighting the need to expand access to genetic counselling services to all patients undergoing genetic testing, for a more comprehensive patient experience. Approximately 36% of the respondents who did undergo testing did not have the support of a counsellor, further underscoring this unmet need. While the oncologist typically initiates discussion on genetic testing, most patients appear to rely on the genetic counsellor for support during the testing process, stressing the need for more genetic counsellors especially in countries where the recognition of genetic counselling as a part of the cancer treatment experience is poor. Enhanced support through counselling will allow patients to have a better understanding and experience of the testing process, leading to greater impact on the patient’s overall treatment outcome. Additionally, genetic counselling may alleviate the stress experienced by a breast cancer patient whose treatment strategy had to be altered following genetic testing, as was reported by more than 40% of the respondents in this survey.

### 4.7 Access to genetic testing is impeded primarily by cost and eligibility criteria

Cost has been identified as a critical barrier in opting for genetic testing and counselling in several studies conducted in the regions that were surveyed in this study (7–9, 11). Similar observations were made in this survey as well. Development of suitable and accessible reimbursement policies is a global need, in countering the financial concerns patients and families have with accessing genetic testing and counselling. While some surveyed countries have devised appropriate reimbursement policies over the years, this survey revealed that most patients in the surveyed countries have poor awareness of the eligibility criteria for reimbursement in their respective countries and may hesitate to access genetic testing or counselling due to a presumption that they may not qualify for reimbursement. Therefore, it is imperative for payers and insurers in the surveyed countries to proactively raise awareness of existing reimbursement schemes and facilitate delivery of these schemes to the patients.

Interestingly, in Australia, where mainstreaming of genetic testing and counselling has removed cost as a major impediment, a considerable proportion of women are unable to get tested due to the extremely stringent eligibility criteria. The Mutational Assessment of newly diagnosed breast cancer using Germline and tumor genomICs (MAGIC) study found that 31 out of 474 study participants were carriers of pathogenic variants in cancer-associated genes (13). However, 18 out of those 31 participants would be ineligible for routine genetic testing per the local testing guidelines (13). Therefore, expansion of local or regional testing policies to all women eligible for genetic testing and counselling as defined by global guidelines such as NCCN is important and may positively impact the treatment experience of breast cancer patients (5).

## 5 Study limitations

A key strength of this study is that it recorded responses from more than 1000 women who have had breast cancer, from diverse backgrounds and experiences of breast cancer genetic testing and counselling. The survey also has its limitations. One such limitation is regional variation in responses, which may have been impacted by inconsistent survey dissemination by the PAGs, depending on the region. Since selection of survey participants was not randomized and may have been impacted by PAG outreach, respondents who had undergone genetic testing were more likely to respond, introducing potential bias in the results. Access to genetic testing may also vary within a region or country due to availability, location in rural and urban areas or due to lack of time, high cost of these services and differences in the complexities in healthcare structure. These variabilities were not accounted for in the analysis. Patients from different breast cancer subtypes were included making some of them ineligible for testing which also poses a potential bias. No pre-specified statistical boundary for significance was established for the survey, which limited statistical analysis of the results.

## 6 Conclusions

To our knowledge, the MAGENTA survey is the first multinational survey conducted for a comprehensive understanding of the breast cancer genetic testing and counselling landscape, from a patient’s perspective. Patients and patient advocates in certain low- and middle-income countries may not have a strong representation in the articulation of the challenges and unmet needs in achieving optimal breast cancer care in their community. We believe that this survey provided patients with a voice, allowing them to communicate their unmet needs to the healthcare professionals, insurer payers and policy makers and enjoy active participation in positively impacting their breast cancer experiences.

Genetic testing appears to be commonly available to patients across the geographies surveyed, although factors such as cost and poor awareness limit universal access to genetic testing and need to be urgently addressed. One of the major gaps as revealed by this survey was the poor uptake of genetic counselling, which remains limited across all the surveyed countries. As this survey demonstrated, low uptake of genetic testing correlated with the absence of genetic counselling in several patient subgroups.

We believe that the findings from this survey will encourage PAGs to drive public awareness programs among patients, healthcare practitioners as well as the public, to showcase the benefits of breast cancer genetic testing and counselling in cancer risk assessment and early treatment decision-making. As also demonstrated by this survey, there is an urgent need to expand the community of genetic counsellors as well as equip surgeons, physicians and oncologists with tools to conduct genetic counselling themselves.

Another important gap highlighted by this survey was the potential role of public policy makers and the government in providing equitable access to these services through appropriate reimbursement policies and cost reduction, as well as incentivizing public and private healthcare institutions to provide these services to breast cancer patients and their families.

We hope that this survey proves to be a breakthrough exercise and transforms the experience of breast cancer patients and their families to drive better patient care.

## Data availability statement

The raw data supporting the conclusions of this article will be made available by the authors, without undue reservation.

## Ethics statement

The findings presented in this article were not based on a study conducted in humans but on a patient survey designed by a patient author steering committee in collaboration with patient advocacy groups from several countries. During the survey, no patients’ personal data were collected. The survey questions were aimed at understanding access and general experiences around breast cancer genetic testing and counseling. Results have been produced and presented in a descriptive manner to demonstrate unmet need and gaps in breast cancer care.

## Author contributions

SP: Writing – review & editing. MA: Writing – review & editing. IB: Writing – review & editing. PG: Writing – review & editing. AH: Writing – review & editing. RK: Writing – review & editing. LK: Writing – review & editing. DR: Writing – review & editing. MS: Writing – review & editing. ES: Writing – review & editing. MY: Writing – review & editing, Methodology, Conceptualization.
