# Supplementary material for: MAGENTA: a Multinational patient survey assessing the Awareness, perceptions and unmet needs in GENetic Testing and counselling among patients with breAst cancer
Source: Front Oncol. 2024 May 14;14:1380349. doi: 10.3389/fonc.2024.1380349 (PMC11130477; doi:10.3389/fonc.2024.1380349)
Supplement: Supplementary file 1 [file DataSheet_1.docx]

Supplementary Material

MAGENTA: A Multinational patient survey assessing the Awareness, perceptions and unmet needs in GENetic Testing and counselling among patients with breAst cancer

**Sarah Powell*, Marta Artigas, Irina Borovova, Poorva Gadiya, Alice Hsu, Ranjit Kaur, Lisa Kidd, Denise Rosenfeld, Mai Mohamed Saeed, Evelin Scarelli8,, Magdy Waheeb Youssef**

***Correspondence:** Corresponding Author: [Sarah@pinkhope.org.au](mailto:Sarah@pinkhope.org.au)

# Supplementary Tables

**Supplementary Table 1. List of patient advocacy groups (PAGs) involved in the MAGENTA survey.**

Patient advocacy groups (PAGs) assisting with localization and dissemination of the survey.

| **Region** | **PAG for survey development & distribution** | **PAGS distributing patient survey** |
| --- | --- | --- |
| **Argentina** | ACIAPO | ACIAPO |
| **Australia** | Pink Hope | Pink Hope |
| **Brazil** | Instituto Oncoguia | Instituto Oncoguia |
| **Egypt** | Together We CANcer | Together We CANcer |
| **India** | NAG Foundation | NAG Foundation |
| **Malaysia** | Breast Cancer Welfare Association Malaysia | SCAN Kuching  Kinabalu Pink Ribbon  Together Against Cancer Association Malaysia  Penang Breast Cancer Hub  Pink Penang Cancer Support Group |
| **Mexico** | Medical and Investigator’s Association Against Cancer | Medical and Investigator’s Association Against Cancer |
| **Russia** | Cancer Patients Association ZDRAVSTVUY | Cancer Patients Association ZDRAVSTVUY |
| **Taiwan** | Formosa Cancer Foundation | Formosa Cancer Foundation |

**Supplementary Table 2. List of questions provided to survey participants.**

The survey was conducted through the Within3 (https://within3.com/) platform and made available to the survey participants. The default number of responses for questions accompanied by response options is 1, with some questions allowing the selection of multiple responses, as specified in the question.

| **No.** | **Question** | **Response options** |
| --- | --- | --- |
| 1 | Which country are you from? | - Argentina - Australia - Brazil - Egypt - India - Malaysia - Mexico - Russia - Taiwan - Other (please specify) |
| 2 | How old are you? Please specify in years. | – |
| 3 | What is your household income? | - Less than USD 30,000 - USD 30,001–USD 60,000 - USD 60,001–USD 90,000 - USD 90,001–USD 120,000 - More than USD 120,000 - I prefer not to disclose |
| 4 | What is your highest level of education? | - Some high school - High school diploma - Some college - Trade/technical/vocational training - Associate’s degree - Bachelor’s degree - Master’s degree - Professional degree - Doctorate |
| 5 | How old were you when you were diagnosed with breast cancer? | – |
| 6 | Have you undergone genetic testing? | - Yes - No |
| 7 | What is your perception of how knowledgeable your doctor is in explaining genetic testing and its implications to you? | - Very knowledgeable - Knowledgeable - Somewhat knowledgeable - Less knowledgeable - Not knowledgeable |
| 8 | What was the role of your oncologist through the genetic testing process? | - My oncologist referred me for genetic testing, explained the process and results to me throughout the process, and used the result to inform treatment selection - My oncologist referred me for genetic testing, explained the process to me at the beginning, and used the result to inform treatment selection - My oncologist referred me for genetic testing and used the result to inform treatment selection - Not applicable - Other (please specify) |
| 9 | How did you feel through the genetic testing process?  Please select up to 3 only. | - Afraid - Angry - Anxious - Ambivalent - Apprehensive - Concerned - Depressed - Grateful - Guilty - Hopeful - Isolated - Overwhelmed - Surprised - Other (please specify) |
| 10 | Do you regret undergoing genetic testing? | - Yes - No |
| 11 | Who was your primary source of information during the genetic testing process? | - Oncologist - Nurse - Genetic counsellor - Patient support groups - Family members - Other (please specify) |
| 12 | Who was your primary source of psychological support during the genetic testing process? | - Oncologist - Nurse - Genetic counsellor - Patient support groups - Family members - Other (please specify) |
| 13 | How did you come to decide to undergo genetic testing? | - After speaking with my doctor/genetic counsellor - After reading up on the internet - After consulting with my family - After another family member was diagnosed - After consulting with patient support groups, including non-governmental organisations, Facebook/social media groups, online forums, etc. - Other (please specify) |
| 14 | How willing would you be to have your children and other family members undergo genetic testing? | - Very willing - Willing - Somewhat willing - Less willing - Not willing |
| 15 | What resources, beyond your oncologist and/or doctor, were available to you to guide your genetic testing experience?  Please select up to 3 only. | - Patient support groups - Genetic counselling - Brochures/pamphlets - Websites - Facebook/social media groups - Other (please specify) |
| 16 | How do you prefer to receive information? Please select up to 3 only. | - Printed materials - Websites - Email - Messaging apps, e.g., WhatsApp, Viber, Line, etc. - Face-to-face - Phone calls - Video chats - Podcasts - Chatbots - Other (please specify) |
| 17 | What was the main reason for deciding against undergoing genetic testing? | - I was not offered genetic testing - I was afraid of what the genetic test results would be - I did not qualify for genetic testing reimbursement - Cost of genetic testing - Life insurance implications - Social stigma from family/community/potential employers - Other (please specify) |
| 18 | Were you offered genetic testing by your doctor? | - Yes - No - I asked for genetic testing |
| 19 | When were you offered genetic testing by your doctor? | - Before diagnosis - At diagnosis - During treatment - After treatment failure - After relapse - I had to ask for genetic testing |
| 20 | Why did you ask for genetic testing? | - I have known family medical history - I have known risk factors - I qualify for genetic testing - I just wanted to know - Other (please specify) |
| 21 | Did you receive any genetic counselling? | - Yes - No - I am not sure |
| 22 | How helpful was genetic counselling in clarifying the genetic testing process and its implications? | - Very helpful - Helpful - Somewhat helpful - Less helpful - Not helpful |
| 23 | How well was the genetic testing process and result implications explained to you by your doctor or genetic counsellor? | - I fully understood the process and implications - I understood most of the process and implications - I understood some of the process and implications - I understood little of the process and implications |
| 24 | Who provided genetic counselling or explained the implications of your genetic testing results to your family? | - My medical oncologist - My surgeon - My GP or other doctor - A genetic counsellor - A clinic nurse - A psychologist - Myself - Nobody - Other (please specify) |
| 25 | Who first raised the discussion of genetic testing and counselling with you? | - My medical oncologist - My surgeon - My GP or other doctor - A genetic counsellor - A clinic nurse - Patient support group - Friend or family member - It was never broached with me - Other (please specify) |
| 26 | What was the outcome of your genetic test? | - *BRCA1*-positive - *BRCA2*-positive - *BRCA*-negative - I have not undergone genetic testing - Other (please specify) |
| 27 | How would you rate the awareness levels of genetic testing and counselling of the following persons and populations?   - Yourself, before diagnosis - Your doctor - Your community | - Very high - High - Moderate - Low - Very low |
| 28 | Did genetic testing change the treatment strategy for your breast cancer? | - Yes - No |
| 29 | How did genetic testing change your treatment strategy?  Select all that apply. | - Unilateral mastectomy to bilateral mastectomy - Chemotherapy/radiotherapy to targeted therapy - Addition of treatment pre- or post-surgery - Addition of surgery pre- or post-treatment - Requiring a second surgery - Other (please specify) |
| 30 | How did the changes in treatment strategy affect you?  Select all that apply. | - Treatment delays due to complexities in switching - Psychological stress - Cost implications - No effect - Other (please specify) |
| 31 | In your opinion, should **all** patients diagnosed with breast cancer undergo genetic testing first before starting treatment? | - Yes - No, only patients with a family history of breast cancer or other risk factors should undergo genetic testing - No |
| 32 | What value(s) do you see/have you experienced with genetic testing?  Please select up to two only. | - To inform treatment decisions - Surveillance and early detection of breast cancer in family members - Awareness for family planning - There is no value - Other (please specify) |
| 33 | Should you qualify, is the cost of **genetic testing** for breast cancer reimbursed in your country? | - Yes, fully - Yes, partially - No - I don’t know |
| 34 | Should you qualify, is the cost of **genetic counselling** for breast cancer reimbursed in your country? | - Yes, fully - Yes, partially - No - I don’t know |
| 35 | Do you have knowledge and understanding of the criteria to qualify for genetic testing in your country? | - Yes - No - I don’t know |
| 36 | In your opinion, how difficult is it to qualify for genetic testing to be reimbursed in your country? | - Very difficult - Difficult - Neither difficult nor easy - Easy - Very easy - I don’t know   Please specify the reason for your answer. |
| 37 | In your opinion, what are the main barriers to genetic testing for you and your family?  Please select up to 2 only. | - Cost - Lack of understanding - Social stigma and discrimination - Family objections - Other (please specify) |
| 38 | In your opinion, what is **a solution that would best address** the gaps and overcome the barriers to genetic testing and counselling in your country? | - Public awareness campaigns to raise patient and community awareness of the value of genetic testing and counselling for breast cancer - Healthcare professional education to increase knowledge and understanding of genetic testing and counselling and its implications in breast cancer treatment - Updating clinical guidelines to include genetic testing and counselling as part of the treatment and management of breast cancer patients - Facilitating the qualification of genetic testing and counselling reimbursement for breast cancer patients - Legislation to protect patients who are genetic mutation carriers from discrimination - Additional educational/informational resources to empower patients and their families on genetic testing and counselling - Other (please specify) |

**Supplementary Table 3. Emotions experienced during genetic testing.**

The percentage responses to the response options to Q09 have been compiled here.

| **Responses** | **n=616 (%)** |
| --- | --- |
| Anxious | 254 (41) |
| Concerned | 128 (21) |
| Grateful | 130 (21) |
| Afraid | 123 (20) |
| Apprehensive | 88 (14) |
| Hopeful | 71 (12) |
| Overwhelmed | 71 (12) |
| Angry | 66 (11) |
| Ambivalent | 63 (10) |
| Depressed | 60 (10) |
| Guilty | 35 (6) |
| Isolated | 56 (9) |
| Surprised | 52 (8) |
| Other | 69 (11) |

#
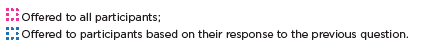

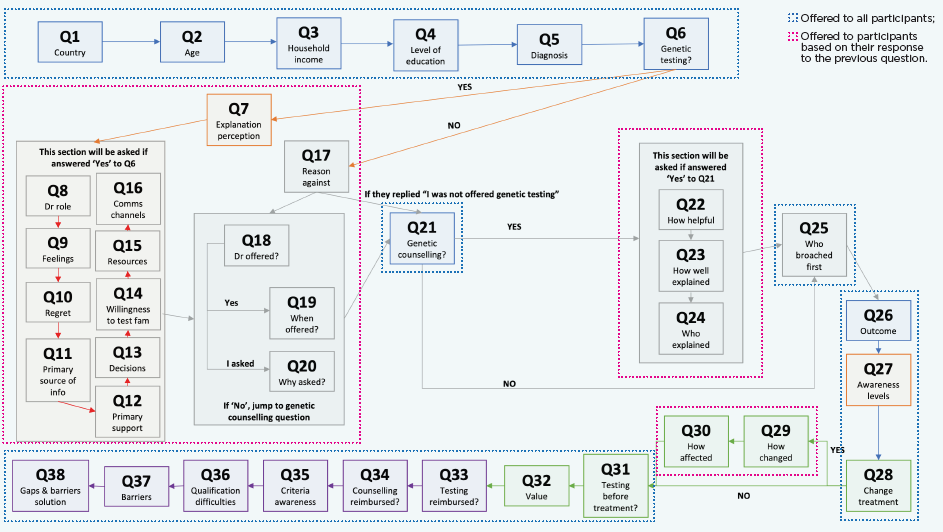
Supplementary Figures

**Supplementary figure 1. Patient survey question sequence.**


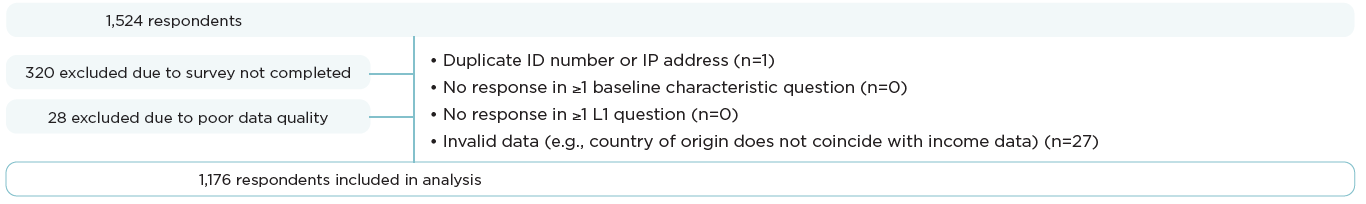
The flowchart shown here demonstrates the sequence of questions encountered by a survey participant. Level 1 questions (boxed in blue) appeared in the survey flow of all participants. Level 2 questions (boxed in pink) appeared in the survey flow, depending on the response to the previous question.

**Supplementary figure 2. Survey population eligibility and analysis.**

The survey was completed by 1,524 respondents with the final analysis set including responses from 1,176 respondents. ID, identification.
